# Management of high-risk patients with hypertension and left ventricular hypertrophy in Germany: differences between cardiac specialists in the inpatient and outpatient setting

**DOI:** 10.1186/1471-2458-6-256

**Published:** 2006-10-19

**Authors:** Heinz Völler, Frank J Sonntag, Joachim Thiery, Karl Wegscheider, Friedrich C Luft, Kurt Bestehorn

**Affiliations:** 1 Klinik am See, Seestrasse 84, D-15562 Rüdersdorf bei Berlin, Germany; 2 Bundesverband Niedergelassener Kardiologen (BNK), Henstedt-Ulzburg, Germany; 3 Institute of Laboratory Medicine, Clinical Chemistry and Molecular Diagnostics, University Hospital, Leipzig, Germany; 4 Institute for Statistics and Econometry, University of Hamburg, Germany; 5 Franz-Volhard Clinical Research Center, Medical Faculty of the Charité, Berlin, Germany; 6 Medical Department, MSD Sharp & Dohme GmbH, Haar, Germany; 7 Institute for Clinical Pharmacology, Technical University, Dresden, Germany

## Abstract

**Background:**

Among patients with hypertension, those with established left ventricular hypertrophy (LVH) represent a high risk cohort with poor prognosis. We aimed to investigate differences in characteristics and health care management of such patients treated as inpatients or outpatients by cardiac specialists.

**Methods:**

Prospective cross-sectional study in patients with hypertension and LVH who were referred to either inpatient care (rehabilitation hospitals) or to outpatient care (cardiology practices).

**Results:**

A total of 6358 inpatients (59.6% males; mean age 66.6 years) and 2246 outpatients (59.5% males; mean age 63.2 years) were followed up for a mean of 23 vs. 52 days, respectively. Inpatients compared to outpatients had a significantly higher prevalence of coronary heart disease, history of stroke, renal failure or diabetes. Mean blood pressure of inpatients compared to outpatients was significantly lower both at entry (150/84 vs. 161/93 mmHg) and at end of follow-up (129/75 vs. 139/83 mmHg). After adjustment for baseline blood pressure and a propensity score, differences between out- and inpatients at end of follow-up were 8.0/5.1 mmHg in favour of inpatients. Blood pressure goals as specified by guidelines were not met by 32% of inpatients and 55% of outpatients.

**Conclusion:**

Inpatients had a higher rate of comorbidities and more advanced atherosclerotic disease than outpatients. Control of hypertension of inpatients was already better on admission than in outpatients, and treatment intensity in this group was also higher during the observation period. While blood pressure lowering was substantial in both groups, there were still a high proportion of patients who did not achieve treatment goals at discharge.

## Background

Cardiovascular (CV) diseases such as coronary heart disease, heart failure and stroke are the leading causes of death in industrialized nations[1,2]. From a public health perspective, it is imperative to address CV risk factors that are amenable to treatment such as life-style adjustments (smoking, obesity), arterial hypertension, lipid disorders, and diabetes mellitus [3]. In recent years, left ventricular hypertrophy (LVH) has emerged as further important risk factor because it indicates target organ damage. Numerous clinical trials have shown that blood pressure reduction to predefined target thresholds reduces LVH, and has substantial influence on subsequent cardiovascular events [4-6]. Against this background, the need for vigorous antihypertensive therapy in these patients in obvious.

In order to promote evidence-based therapy for hypertension, a number of guidelines have been issued by national and international societies, [7-9] and such guidelines are accepted as standards of care in most countries. Nevertheless, physician behaviour is not necessarily strongly influenced by these recommendations [10]. Data from several countries, including Germany, document that only about two-thirds of known hypertensive individuals receive any treatment and less than half of these patients are controlled to target values [11-13].

These studies addressed the situation in the community or in the primary care setting. Specialist care might be better than care from general practitioners [14]. The German health care system provides inpatient rehabilitation for patients who have documented target organ damage, particularly after having experienced a CV event such as acute coronary syndrome (with or without interventional or surgical revascularization) or after such an elective intervention without a previous acute event. Usually acute care hospitals refer patients to rehabilitation centers. The aim of rehabilitation is to optimize the patients' CV risk profile and to provide education in terms of life-style interventions [15].

Another option for specialist care is provided in an outpatient setting. Primary care physicians may refer patients with high CV risk – independent of a previous CV event – to a cardiac specialist in an outpatient setting for diagnostic and therapeutic support and risk stratification.

To date it has not been investigated whether patients referred to cardiac specialists in an inpatient versus an outpatient setting differ from each other in terms of demographics or comorbidities, are treated differently, or have different outcomes. Therefore we addressed these questions in a large-scale prospective study. We focused on hypertensive patients with comorbid LVH as high risk group easily identified by cardiac specialists, with high prevalence and thus substantial public health implication.

## Methods

### Study design and patients

This was an observational epidemiological prospective study (Risk Factors in Hypertension Registry, RIKHY), performed between September 2002 and January 2003 in a convenience sample of cardiac experts throughout Germany. A total of 251 certified cardiologists (treating outpatients only) participated, as well as 100 rehabilitation centers that specialized on the management of patients with CV diseases (and recruiting inpatients only). They documented a total of 12 273 patients who met the entry criteria. Of those, 7 095 (58%) were from inpatient facilities and 5 178 (42%) were from outpatient practices.

Male and female patients were eligible for inclusion in the present analysis, if they were aged 18 years or older, had a physician diagnosis of arterial hypertension and concomitant LVH, provided informed consent and if their clinical follow-up duration was scheduled to be at least 3 days. LVH was assessed according to standard criteria in the electrocardiogram (using the Lewis, Sokolow-Lyon and/or Cornell indices), in the echocardiography or both [16-20]. No formal exclusion criteria were established to document as closely as possible the typical patient pattern in this setting. The study was approved by the certified ethics committee of the Bavarian Physicians Chamber.

### Assessments

Two visits were foreseen, on admission and at discharge, to document patient data on case report forms. The schedule according to which outpatients were followed up was not regulated in the study protocol but left to the physicians in order to demonstrate real life patient care. Physicians in both settings recorded demographic data, medical history, details on cardiovascular risk factors and comorbidities, results of physical examination, laboratory and procedural test results, medications and the dosages of their patients. Reasons for the initiation of antihypertensive drugs and any changes in medications were noted. Blood pressure was recorded by trained nurses with a certified device on admission and at discharge with the patient sitting for at least 3 minutes. The blood pressure at the fifth Korotkoff sound was taken as the diastolic pressure. Heart failure was diagnosed with standard clinical methods detailed elsewhere [21]. The doctor's clinical assessment of each individual patient by a standardized questionnaire included indicating the presence or absence of further predefined concomitant diseases (such as renal dysfunction, pulmonary disease etc.).

### Data management and statistics

Data were stored in a Microsoft Access 97 database and analyzed with the SAS statistical program (release 8.2), SPSS (release 13.0) or SYSTAT (release 11.0), respectively. Descriptive statistics were derived and comparisons between inpatients and outpatients were conducted by t-tests or chi square tests as appropriate. Significance was accepted at the p < 0.05 level. Furthermore, local regression lines (LOWESS regressions)[22] were computed for a baseline-adjusted comparison of changes in systolic blood pressure between groups, corrected for reliability to avoid the regression-towards-the-mean effect. To cope with a potential referral bias, a propensity analysis was performed using a stepwise logistic regression approach that was based on all available baseline variables [23,24]. Finally, to account for referral effects and hospital- or practice-related cluster effects, adjusted discharge blood pressure differences were calculated using a hierarchical model including the blood pressure at admission and the propensity score as fixed covariates and facilities as random effect.

## Results

### Demographic data and distribution of risk factors and target organ damage

8604 of the recruited patients met the hypertension/LVH criteria, namely 6358 (74%) inpatients and 2246 (26%) outpatients. In terms of demographics, there were no major differences between groups. Inpatients had a mean age of 66.6 (± 12.1) years, and 59.6% were male; outpatients were 63.2 (± 11.3) years of age; and 59.7% were male. In both cohorts, mean BMI was 28.2 kg/m^2^.

**Figure 1:**
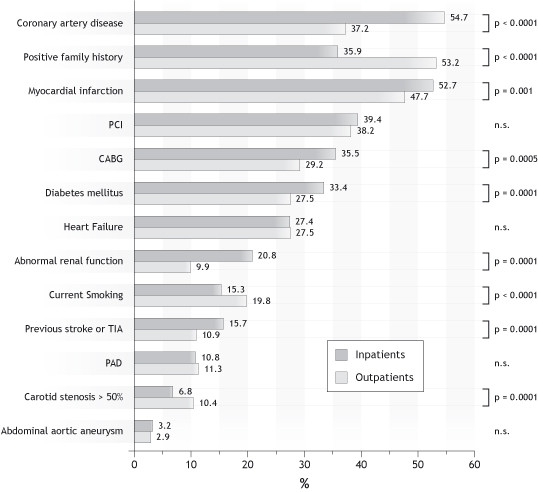
**Comorbidities and risk factors in inpatients and outpatients on admission**. Coronary artery disease includes one or more of the following: myocardial infarction, percutaneous coronary intervention (PCI), coronary artery bypass graft (CABG). PAD, peripheral arterial disease.

Almost all patients (99%) were currently hypertensive and all had LVH. In 70%, LVH diagnosis was established with echocardiography, in 8% with electrocardiography, and in the remainder with both procedures. Additional risk factors, target organ damage and atherosclerotic manifestations were highly prevalent in both groups, as presented in Figure 1. The most frequent comorbidity was coronary artery disease, followed by diabetes mellitus, heart failure and abnormal renal function. There were significant differences in the proportions of comorbidities between the cohorts: inpatients had significantly higher rates of coronary artery disease, previous stroke or TIA, abnormal renal function, and diabetes. Conversely, outpatients had higher rates of carotid stenosis.

Twenty-two baseline characteristics contributed to the propensity score. Of these, 4 variables accounted for 85% of the variation in the propensity score: abnormal renal function, diastolic and systolic blood pressure and family history for hypertension. After adjustment for the score, 21 out of the 22 baseline variables were not significantly different between groups, indicating that the propensity score explained the referral effects rather well.

### Duration of follow-up

Inpatients were discharged after a mean of 23 days (3.3 weeks), outpatients were followed up over a mean of 52 days (7.4 weeks), and then referred back to primary care.

### Blood Pressure

**Figure 2:**
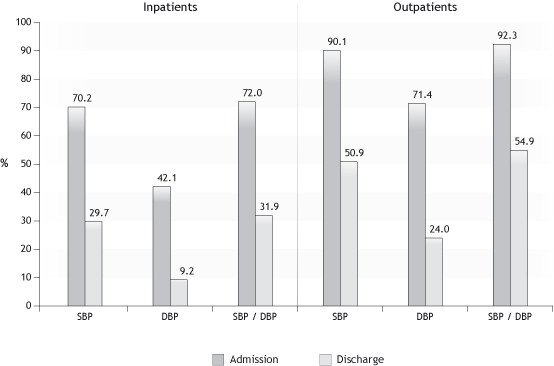
**Missed BP goals: Percentages of patients with systolic BP ≥ 140 mmHg and/or diastolic BP ≥ 90 mmHg at discharge**. DBP, diastolic blood pressure; SBP, systolic blood pressure.

Mean blood pressure (BP) values on admission was 150.5/84.4 mmHg for inpatients and thus substantially lower than in outpatients (160.6/92.7 mmHg, difference between groups p < 0.0001). BP goals at admission (defined as systolic/diastolic BP < 140/90) were not met in 72% of inpatients and 92% of outpatients, respectively, as displayed in Figure 2.

**Figure 3:**
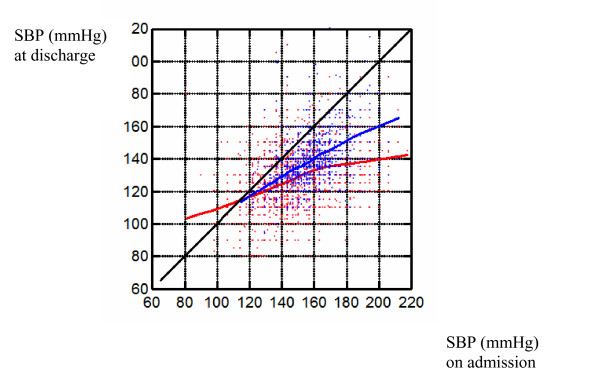
**Change of systolic blood pressure from admission (abscissa) to discharge (ordinate) in inpatients and outpatients**. LOWESS regression. Inpatients are represented by the red regression line, the outpatient values by the blue regression line. Values are corrected for reliability (i.e., for regression-to-the-mean effects).

Figure 3 displays a LOWESS regression analysis of the results of the antihypertensive treatment in inpatients and outpatients. The regression lines between groups diverge with increasing SBP admission levels. Thus, at 140 mmHg, the difference was 4.5, at 150 mmHg 6.4, at 160 mmHg 7.3 and at 180 mmHg 11 mmHg in favour of the inpatients. The slight bow of the red line in inpatients (at ca. 160 mmHg) marks an assumed threshold of intensified antihypertensive medication whereas the blue line of the outpatients remains straight. At the end of follow-up mean blood pressure was 128.7/75.3 mmHg (difference -21.8/-9.1 mmHg in inpatients) and 139.2/82.9 mmHg (-21.4/-9.8 mmHg in outpatients). The proportion of patients with uncontrolled PB was reduced, yet was still high in absolute terms in both groups (32% in inpatients and 55% in outpatients, p < 0.0001; Figure 2). The apparent 10.2/7.6 mmHg difference between inpatients and outpatients was only slightly reduced to 8.0/5.1 mmHg after adjustment for baseline and remained highly significant (p < 0.0001/p < 0.0001) even after correction for cluster effects.

### Antihypertensive medication

**Figure 4:**
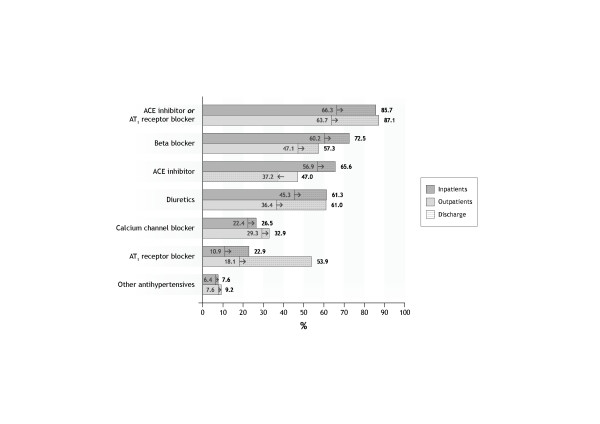
**Antihypertensive medication on admission and at discharge**. Figure displays the percentages of antihypertensives by class in inpatients and outpatients at admission and discharge. Arrows indicate whether the drugs are more or less frequently used at discharge. → denotes *more *frequently prescribed at discharge compared to admission. ← denotes *less *frequently prescribed at discharge compared to admission

Drug treatment rates increased between the beginning and end of the survey from 91% to 98% in inpatients and from 88% to 95% in outpatients. Figure 4 displays the antihypertensive medication of inpatients and outpatients on admission and at discharge. The mean/median number of drugs on admission was not significantly different in inpatients (2.0/2) and outpatients (1.9/2). In both groups, mean number of drugs was increased to 2.6/3 or 2.5/3, respectively.

On admission, conventional drugs (beta blockers, ACE inhibitors, calcium channel blockers and diuretics) were most frequently prescribed. Usage of all drug classes was increased at discharge, with the exception of ACE inhibitors that were reduced in outpatients. However, this was compensated for by a substantial increase in AT_1 _blockers in this group. In total, the prescription rates of inhibitors of the renin-angiotensin system were increased in both groups at discharge.

Numerous medication changes were made in both individual inpatients and outpatients, and in only 25% (inpatients) and 21% (outpatients) medications at entry were maintained without any changes. Table 1 shows the reasons for medication changes as reported by physicians and patients. Inadequate response to treatment was the most frequent reason given. In outpatients this reason was significantly more frequently stated, as were reasons related to tolerability and medication compliance. Side effects were reported in substantial proportions of patients (7% in inpatients and 21% in outpatients). Cough was reported in a third of inpatients and two thirds of outpatients that were intolerant to ACE inhibitors. In both groups, angioedema were reported (26% and 21% of those patients who discontinued ACE inhibitors).

## Discussion

The present study was conducted to assess whether high-risk patients (hypertensive with LVH) treated by cardiac specialists in the community setting (outpatients) differ from patients treated by specialists in cardiac rehabilitation centers (inpatients) in terms of patient characteristics and comorbidities, process of care, and clinical outcomes. The principle findings of the study are as follows: First, inpatients had a higher rate of comorbidities and more advanced atherosclerotic disease. Second, control of hypertension of inpatients was already better on admission than in outpatients, and treatment intensity in this group was also higher during the observation period. Third, while blood pressure lowering was substantial in both groups, there were still a high proportion of patients that did not achieve treatment goals at discharge.

Current guidelines for the management of hypertension such as the one of the European Hypertension Society/European Society of Cardiology stress the importance of searching for comorbidities ("associated clinical conditions" and "target organ damage"), as they substantially influence prognosis of the patient [8]. These associated clinical conditions (cerebrovascular disease, heart disease, renal disease, peripheral vascular disease and advanced retinopathy) or typical forms of target organ damage (LVH, arterial wall thickening e.g. in the carotids, or atherosclerotic plaques, nephropathy or microalbuminuria) have been clearly linked with elevated risk in epidemiological studies. Either of these findings puts the patient at a risk which is at least as high as in diabetes mellitus, or matches the combined presence of at least 3 conventional risk factors (such as higher age, smoking, dyslipidemia, abdominal obesity, family history of premature cardiovascular disease). Depending on the level of blood pressure, such a patient is at least at "high added risk" [8].

The present study focused primarily on LVH as this condition in cardiac specialist care, when diagnosed, should trigger intensive blood lowering treatment. This approach is clearly evidence-based, as a number of studies have documented substantial, however variably strong, regression of LVH with various antihypertensive drugs [5]. The large-scale, long-term LIFE study with losartan is of particular interest as it showed, in line with Framingham [18] and HOPE [25] outcomes, that the greater regression of LVH was paralleled by a reduced incidence of CV events [6]. Mainly based on this study, the ESC/ISH guidelines explicitly recommend AT_1 _blockers in patients with LVH [8].

Our study found that hypertensive patients with LVH when referred as inpatients were generally sicker than outpatients when taking into account comorbidities (especially in view of atherosclerotic complications). Obviously, referring primary care physicians trust that these patients will benefit from the characteristics of an inpatient setting (off-work atmosphere, additional educational elements, and generally more comprehensive treatment options).

Regarding general hypertension management and medication choice, cardiac specialists treating inpatients or outpatients seemed to follow guideline recommendations to a substantial extent. Antihypertensive treatment during the observation was intensified, as evidenced by the increased proportion of medically treated patients, by the increased number of drugs (2.5 at the discharge), and the preference of inhibitors of the renin angiotensin system. The LOWESS regression suggests that inpatients were somewhat more aggressively treated than outpatients; however, this was seen only in patients with SBP values above 160 mmHg. Notably, AT_1 _receptor blockers were much more frequently used in outpatients than in inpatients (54% versus 37%), whereas the opposite held true for ACE inhibitors (66% versus 37%). Potential reasons for this difference might include cost considerations. Interestingly outpatients seemed more difficult to manage, as they had much higher medication switch rates with lack of tolerability being three-fold increased compared to inpatients, and lack of compliance being substantially increased. It is known from the controlled study setting [26] as well as in primary care that antihypertensive medication changes due to a variety of reasons are the rule rather than the exception [27,28]. All classes seem to have similar rates of non-response among patients, however, the newer drugs such as AT_1 _blockers seem to be associated under study as well as clinical practice conditions with better tolerability and consequently, higher persistence rates among treated patients [27,29]. This might be an explanation why AT_1 _receptor blockers were preferred in outpatients. Nonetheless, previous reports from the primary care setting suggest that there is wide-spread reluctance of physicians to treat hypertension aggressively enough. Underlying reasons might be, at least in the elderly, the fear of doing harm by applying too-intensive treatment [30], and as noted in our study, compliance problems of patients if side effects are experienced [31]. "Clinical inertia", a term that summarizes three related problems associated with inadequate management of chronic diseases (overestimation of care provided; use of 'soft' reasons to avoid intensification of therapy; and lack of education, training and practice organisation aimed at achieving treatment goals)[32], may also play a major role, as has recently been suggested as a reason for the suboptimal hypertension treatment in the primary care sector in Germany [33]. Reimbursement issues in Germany at least in the outpatient setting (fixed budget system [34]) may also contribute to underprescribing and undertreatment.

In terms of treatment outcomes, the mean absolute BP lowering effect achieved was substantial in both groups (SBP -22/-21 mmHg, DBP -9/-10 mmHg). In a recent metaanalysis of 354 randomised controlled trials including all current first-line antihypertensives, the mean BP lowering effect across all drug classes in the standard doses was SBP/DBP -9.1/-5.5 mmHg [29]. Thus, even when accounting for the placebo effect which adds to the drug effect, the BP reduction achieved by cardiac specialists in our study was not inferior to that achieved under highly controlled study conditions. Further, they managed pre-treated patients, with the need to switch or add antihypertensive drugs, and had only a limited follow-up period to identify an optimized treatment for their patients. The mean average number of 2.5 drugs in both groups at discharge was still below the average of other observations and clinical studies to reach BP goals, where up to 5 different agents were needed [35]. This is especially the case in patients with diabetes or nephropathy [36,37], which made up a substantial fraction of individuals in both inpatients and outpatients in our study.

The general BP target of <140/90 mmHg were achieved by inpatients more frequently than by outpatients. This might be due to the fact that outpatient practitioners had to manage higher blood pressures at entry. However, even with comparable baseline values, in the inpatient setting more pronounced blood pressure reductions were achieved in the outpatient setting. While control rates as such in both groups were suboptimal, it has to be stressed that physicians had to treat "difficult" patients with multiple comorbidities within the constrictions of a challenging time frame.

The present study was not designed to answer the question whether cardiac specialists in the hospital setting compared to those in the community setting provide better care for patients. A number of studies compared certified cardiologist care with internists or primary care physicians, and found improved care for cardiology conditions, mainly in the treatment of patients with acute myocardial infarction or heart failure [38-43]. However, differences are multifactorial, and often a function of study design or patient selection [39]. Treatment initiation in a hospital setting has been reported to be especially effective for cardioprotective therapies [44]. As patients referred to rehabilitation centers usually have been pre-treated in acute care hospitals, they might benefit from better cardiac management. In terms of hypertension treatment, our study supports this view, because inpatients seemed to receive more intensive care. Further, expert physicians credentialed as "hypertension specialists" (by the German Hypertension League, similarly to the American Society of Hypertension Specialists Program [45]) were not identified nor were we able to analyse their treatment approaches.

## Conclusion

We found that high-risk hypertensive patients with LVH treated as inpatients versus as outpatients differed in terms of their profiles, and comorbidities. While their blood pressure treatment is intensified by cardiac specialists in both settings, there is still substantial room for improvement in blood pressure and corresponding risk reduction. There was a substantial gap in blood pressure control rates to published targets. This data confirm the difficulties reported in achieving treatment goals in other settings and indications. As the results are more than three years old, a similar study should be initiated shortly to assess whether the situation has improved.

## Competing interests

KB is as Director of Medical Research employed by MSD Sharp&Dohme GmbH, Haar, Germany, and is also affiliated with the Institute for Clinical Pharmacology, Technical University of Dresden. All other authors declare that they have no competing interests. MSD funded the study with an educational grant and covered the publication costs.

## Authors' contributions

HV and KB designed the study, SF, JT, and FCL were advisors to study design and interpretation of the study, KW was the statistical advisor and performed all analyses. KB and HV wrote the first draft of the manuscript, and all authors contributed to the revisions and gave their consent to the final manuscript.

## Pre-publication history

The pre-publication history for this paper can be accessed here:



## Figures and Tables

**Table 1:** Reasons for changing medications

**Reason**	**Inpatients **(N = 6358) **%**	**Outpatients **(N = 2246) **%**	**p value**
Lack of efficacy	58.6	67.7	<0.0001
Not tolerated*	7.3	21.1	<0.0001
Cough (with ACE inhibitors)	35.3	62.1	<0.0001
Angioedema	25.8	20.6	n.s.
Others	35.7	20.4	<0.0001
Not reported	7.5	8.2	n.s.
Contraindication	1.7	1.3	n.s
Drug-drug interaction	1.0	0.6	n.s
Compliance	2.7	4.2	n.s
Patient's request	2.9	5.3	<0.0001
Other reasons	14	6.7	<0.0001
**No change**	25.4	20.8	<0.0001

n.s., not significant

* Several reasons may apply in an individual patient.
